# Mitochondrial genetics – new model uncovering roles in tumorigenicity and metastasis

**DOI:** 10.18632/oncoscience.407

**Published:** 2018-04-29

**Authors:** Thomas C. Beadnell, Danny R. Welch

**Affiliations:** Department of Cancer Biology, The Kansas University Medical Center, and The University of Kansas Cancer Center, Kansas City, KS 66160, USA

**Keywords:** mitochondria, genetics, metastasis, tumorigenicity

Why do some patients develop cancer, or why do some patients respond to therapy, while others do not? The underlying genetics are beginning to provide insights into these critically important questions and may eventually guide physicians toward more effective prevention and treatments. For example, Kent Hunter and colleagues demonstrated that specific single nucleotide polymorphisms (SNP) play important roles in metastasis efficiency [1]. They crossed male FVB/NJ-TgN (MMTV- PyMT)634nul (PyMT) mice to female mice from multiple strains and mapped nuclear genes that control metastasis. Their experimental design, however, also included an alternative explanation, since the non-transgenic mice were female: i.e., maternally-inherited mitochondrial genome could also encode metastasis modifier loci.

The mitochondrial genome (~16,300 base pairs encoding 37 genes (13 electron transport proteins, 22 tRNA and 2 rRNA). Mitochondrial DNA (mtDNA) is maternally inherited and mtDNA SNP are frequently utilized to map ancestry as they provide a snapshot of common lineage in both mice and humans. Since most research mice are inbred there is minimal genetic variability amongst the strains. Low variability decreases the number of confounding variables, providing a cleaner model for understanding the role of mtDNA SNP in regulating disease susceptibility.

To study the roles of mitochondrial genetics in complex phenotypes, including metastasis, we developed the mitochondrial nuclear exchange (MNX) mouse in which pronuclei from embryos were implanted into enucleated embryos to generate a reconstructed zygote harboring non-syngeneic nuclear and mitochondrial DNA. Embryos are then implanted into pseudopregnant mice, genotyped, and then bred to create colonies [2, 3]. MNX mice have clear advantages over other models used to study the impact of mitochondrial genetics as they avoid the time associated with generation of conplastic strains and the genetic drift that occurs over multi-generational backcrossing. Also, the MNX mice are never exposed to DNA mutagens often used to deplete mitochondrial DNA in the Rho-null and cybrid models.

Feeley *et al*. demonstrated that mtDNA polymorphisms impact mammary tumor latency as well as the severity of metastatic disease in the PyMT metastatic breast cancer model in parallel with the Hunter study. Briefly, PyMT mice were bred to MNX mice with syngeneic (FVB) nuclear genomes paired with FVB (FF), C57BL/6J (FC) or BALB/cJ (FB) mtDNA. Tumor latency and lung metastasis were impacted almost identically to the findings reported by Hunter and colleagues. That is: C57BL/6J mtDNA lengthened latency of first tumor development and BALB/cJ shortened latency while reducing and promoting lung colony size, respectively [4]. Brinker et al. extended the Feeley study, except with FVB mice over-expressing the HER2 oncogene crossed with MNX strains [5]. Again, mtDNA altered mammary tumor latency and metastasis. However, both FC and FB mice reduced the number of metastases while increasing metastasis size. The results clearly demonstrated that mammary tumor latency and metastasis are affected by cooperation between oncogenic drivers and mtDNA polymorphisms.

Additionally, MNX mice have also been used to demonstrate that mitochondrial polymorphisms are not restricted to modifying cancer susceptibility. Fetterman *et al*. demonstrated that mitochondrial polymorphisms in the C57BL/6J mice lead to altered susceptibility to atherosclerosis and cardiac stress in comparison to C3H/ HeN mtDNA backgrounds [2].

Collectively, the results from these experiments highlight the importance of nuclear-mitochondrial cross- talk in complex diseases. Since cancer and atherosclerosis require coordinated expression of multiple genes, Vivian *et al*. analyzed mitochondria-mediated changes in nuclear DNA methylation and gene expression by comparing wild-type and MNX brain tissues [6]. While metabolic differences would expectedly alter metabolite pools required for methylation [2] and -- by extension, the ability of cells to methylate DNA -- their data clearly demonstrated that mtDNA selectively changed where the epigenetic marks were altered. The underlying controlling mechanisms are an area of intense current research.

Mitochondrial genetic diseases have been described [7], although they are relatively rare. Their roles in more complex diseases as quantitative trait loci [8] are only recently becoming more appreciated. Due to the mitochondria's modest size, deeper understanding of the cross-talk between mtDNA and nDNA may provide insights that address disease susceptibility and progression as well as responses to therapy.

## References

[1] Lifsted T (1998). Int J Cancer.

[2] Fetterman JL (2013). Biochem J.

[3] Kesterson RA (2016). Bio Protoc.

[4] Feeley KP (2015). Cancer Res.

[5] Brinker AE (2017). Cancer Res.

[6] Vivian CJ (2017). Cancer Res.

[7] Tuppen HA (2010). Biochim Biophys Acta.

[8] Bussard KM, Siracusa LD (2017). Cancer Res.

